# Identifying Truly HPV-Driven Head and Neck Squamous Cell Carcinoma by QuantiGene-Molecular-Profiling-Histology Assay Allows for More Precise Prognosis Prediction

**DOI:** 10.3390/ijms252413643

**Published:** 2024-12-20

**Authors:** Lili Liang, Andreas E. Albers, Eliane T. Taube, Jonathan Pohl, Stephanie Schmidt, Marla Greve, Andreas M. Kaufmann

**Affiliations:** 1 HPV Research Laboratory, Department for Gynecology, Charité—Universitätsmedizin Berlin, Corporate Member of Freie Universität Berlin and Humboldt-Universität zu Berlin, Augustenburger Platz 1, 13353 Berlin, Germany; lili.liang@charite.de (L.L.);; 2 Klinische Medizin, Schwerpunkt Hals-Nasen-Ohren-Heilkunde, MSB Medical School Berlin, Hochschule für Gesundheit und Medizin, Rüdesheimerstr. 50, 14197 Berlin, Germany; 3 Institute for Pathology, Charité—Universitätsmedizin Berlin, Corporate Member of Freie Universität Berlin and Humboldt-Universität zu Berlin, Charitéplatz 1, 10117 Berlin, Germany

**Keywords:** persistent HPV infection, HPV DNA, molecular diagnosis, mRNA quantification, biomarkers, chemo-radiation therapy

## Abstract

Head and neck squamous cell carcinoma (HNSCC) with discordant diagnostic patterns of HPV^+^/p16^−^ or HPV^−^/p16^+^ correlate with worse prognosis. This study aims to identify truly HPV-driven HNSCCs using a QuantiGene-Molecular-Profiling-Histology (QG-MPH) assay for identifying transcriptionally active HPV. Of 97 FFPE samples analyzed, 68 were valid for HPV DNA detection by PCR and quantification of HPV E7 and p16 ^INK4a^ mRNA by QG-MPH. p16 ^INK4a^ mRNA expression was compared with p16 protein expression via immunohistochemistry (p16 IHC). Among the 68 cases, 26 (38.2%) showed increased high-risk HPV E7 mRNA expression (hrHPV E7 mRNA^+^), while 37 (54.4%) were hrHPV DNA^+^. Concordance between HPV DNA and mRNA status was 70.1%. Notably, 79.2% of E7 mRNA^+^ cases were p16 IHC^+^, compared to 55.9% of DNA^+^ cases, demonstrating better concordance between HPV E7 mRNA^+^ status and p16 mRNA expression plus p16 IHC positivity. All patients (19/19) in the HPV E7 mRNA^+^/p16 IHC^+^ group survived the 5-year follow-up, compared to 59.5% (22/37) in the HPV E7 mRNA^−^/p16 IHC^−^ group (*p* = 0.001). Specifically, the OS rate was 57.1% (8/14) in the group with discordant HPV DNA and p16 IHC results, compared to 40% (3/5) in the group with discordant HPV E7 mRNA and p16 IHC results. These findings highlight the better outcomes for the transcriptionally active HPV cases and indicate the prognostic disadvantage for patients with discordant patterns and the advantages for incorporating the molecular mRNA profiling by QG-MPH to p16 IHC. In conclusion, QG-MPH quantification of E7 and p16 ^INK4a^ mRNA more precisely identifies truly HPV-driven from non-HPV-driven HNSCC, compared to HPV DNA testing alone or with p16 IHC, which reduces misclassification and provides valuable implications for improved prognosis prediction and therapeutic decision-making.

## 1. Introduction

Head and neck squamous cell carcinoma (HNSCC) is a heterogeneous group of tumors that arise from the squamous cell epithelium of the oral cavity, pharynx, and larynx [1,2]. There are an estimated 890,000 new cases and 450,000 deaths worldwide each year [3,4]. The incidence of HNSCC is expected to increase by 30% by 2030 globally, that is, 1.08 million new cases annually [5]. In addition to well-known risk factors, such as alcoholic beverages and tobacco products, Human Papillomavirus (HPV)-attributable HNSCCs continue to rise in many countries [4]. Notably, high-risk HPV (hrHPV), is increasingly recognized as a key etiological factor in the initiation and development of HNSCC, primarily oropharyngeal squamous cell carcinoma (OPSCC) [5]. Globally, around 33% of OPSCC cases were attributed to HPV in 2021, which mainly involves cancers of tonsils and the base of tongue [2,6]. Importantly, HPV-attributable HNSCCs continue to rise in many countries [4]. Among the HPV subtypes, HPV 16 is responsible for up to 90–95% of HPV DNA-positive (HPV DNA^+^) HNSCC [7], followed by HPV18, 31, and 33 [2,8]. HPV DNA^+^ HNSCC presents different clinicopathological features, such as better susceptibility for chemoradiation and more favorable outcomes, compared to the HPV DNA-negative (HPV DNA^−^) malignancies [5,7,9].

Testing for hrHPV DNA using polymerase chain reaction (PCR)-based techniques has facilitated diagnosis of HPV-associated cancers. However, HPV DNA positivity alone does not definitively prove that the cancer is truly HPV-driven, as the virus may exist in a transcriptionally silent state. Consequently, HPV mRNA pattern analysis is considered a more meaningful and reliable method for identifying HPV-driven cancers [10,11]. Understanding HPV viral transcriptional activity in HNSCC is crucial for determining whether HPV serves as a true disease driver. During a truly HPV-driven carcinogenesis, the viral oncogene E7 is continuously expressed, suggesting a transcriptionally active HPV infection. This activity leads to the upregulation of p16 ^INK4a^ mRNA and the subsequent overexpression of the p16 protein, which can be detectable by immunohistochemistry staining (referred to herein as p16 IHC) [5,12]. In contrast, transcriptionally silent HPV infections lack E7 mRNA expression, resulting in the absence of both p16 mRNA and p16 protein [5,12]. Accordingly, p16 IHC has been suggested as a surrogate marker for HPV status in TNM-8 staging system [13]. However, discordant patterns, including HPV DNA-positive but p16 IHC-negative (indicated as HPV DNA^+^/p16 IHC^−^) and HPV DNA-negative but p16 IHC-positive (indicated as HPV DNA^−^/p16 IHC^+^) cases, have also been observed in HNSCC [14,15]. Evidence indicates that patients with these discordant patterns have a worse prognosis, including a lower 5-year overall survival (OS) rate, compared to patients with both HPV DNA^+^ and p16 IHC^+^ (indicated as HPV DNA^+^/p16 IHC^+^) [5,16,17]. The underlying causes of these discordances remain unclear, particularly whether p16 IHC-negative expression in HPV DNA^+^ cases results from transcriptionally silent HPV infection and lack of E7 transcripts or whether p16 IHC positivity in the HPV DNA^−^ cases is induced by a non-HPV-dependent pathway. Therefore, p16 IHC-positive expression is not a perfect surrogate for HPV etiological diagnosis. The combination of HPV DNA testing and p16 IHC may still overestimate HPV’s carcinogenic role in some cases, as transcriptionally silent HPV infection is not necessarily related to carcinogenesis [5]. In current clinical practice, patients with HPV DNA^+^/p16 IHC^−^ or HPV DNA^−^/p16 IHC^+^ results often receive the same standard treatment as those with HPV-driven tumors. Meanwhile, some clinical trials are exploring the potential benefit of treatment de-escalation for patients with HPV-positive cancer [18,19]. Misclassification of truly HPV-driven and pseudo-HPV-driven HNSCC due to discordant HPV DNA and p16 IHC patterns raises concerns about whether all HPV DNA^+^ and/or p16 IHC^+^ tumors are genuinely HPV-driven. Patients whose TNM-8 staging is down-staged based on the HPV DNA status may be at risk of undertreatment, potentially leading to adverse outcomes. Therefore, it is urgent to advocate for the accurate identification of truly HPV-driven HNSCC to ensure proper interpretation of clinical data and to guide therapeutic strategy and prognosis predictions effectively.

In response to the urgent need to precisely characterize the etiological role of HPV in HNSCC, we propose to elucidate true HPV-driven carcinogenesis by a comprehensive approach of molecular expression profiling. In this study, we describe the application of the novel QuantiGene-Molecular-Profiling-Histology (QG-MPH) assay, which quantifies mRNA expression by multiplexed measurement of 18 individual HPV oncogene E7 plus HPV 16E6, p16 ^INK4a^ (referred to herein as p16), and other cancer-associated cellular biomarkers. The approach aims to improve the etiological diagnosis of HNSCC, evaluating the prognostic value of combining HPV mRNA testing with p16 mRNA and p16 IHC, with potential implication for therapeutic decision and prognostic assessment.

## 2. Results

### 2.1. Clinical Characteristics

A total of 68 cases with valid HPV DNA and mRNA test results were included in the statistical analysis (Table 1). The mean age at diagnosis was 60.8 ± 10.1 years. Of these patients, 28 (41.2%) had a healthy weight (BMI 18.5–24.9), while 25 (36.8%) were classified as overweight. A history of smoking was found in 48 (70.6%) patients. The most common tumor sites were the tonsil (n = 25, 36.8%), floor of the mouth (n = 14, 20.6%), and base of tongue (n = 11, 16.2%). Tumor staging, revised according to the AJCC 8th edition guidelines, was distributed as follows: Stage I (n = 16, 23.5%), Stage II (n = 26, 38.2%), Stage III (n = 12, 17.6%), and Stage IV (n = 14, 20.6%). The tumor differentiation of specimens from the initial surgery was diagnosed: Grade II (n = 33, 48.5%) and Grade III (n = 33, 48.5%), and only two (2.9%) cases were Grade I. These patients had neither prior HPV testing nor p16 IHC when diagnosed.

Table 2 summarizes the clinicopathological features stratified by HPV16 mRNA status. There were no statistically significant differences between the HPV16 mRNA-positive and -negative group in terms of mean age, tumor staging and grade, pN status, or the proportion of receiving postoperative radiation. Notably, the HPV16 mRNA^+^ group had statistically significantly fewer patients with advanced tumor stage and a lower proportion receiving postoperative chemotherapy compared to the HPV16 mRNA^−^ group (*p* = 0.010, *p* = 0.004, respectively).

### 2.2. HPV Prevalence by HPV DNA and mRNA Status

The HPV prevalence by HPV DNA and mRNA status is summarized in Table 3. Generally, out of 68 cases, 37 (54.4%) tested hrHPV DNA^+^, including 34 (50.0%) HPV16 DNA^+^ cases. The majority was single HPV infection (n = 32), with two cases showing multiple infections (HPV16^+^/18^+^ or HPV16^+^/35^+^). There were a few other hrHPV-infected cases detected: two HPV33^+^ cases and one HPV18^+^/82^+^ case. The QuantiGene-Molecular-Profiling-Histology (QG-MPH) assay detected positive hrHPV E7 mRNA expression (hrHPV E7 mRNA^+^) in 26 (38.2%) cases, with 24 (35.3%) specifically positive for HPV16 (HPV16 E7 mRNA^+^). Additionally, HPV18 E7 mRNA^+^ and HPV33 E7 mRNA^+^ were found (one case each). The concordance between HPV DNA and mRNA status was calculated. In total, 70.1% (26/37) of hrHPV DNA^+^ cases were hrHPV mRNA^+^. For HPV16 specifically, 70.5% (24/34) of HPV16 DNA^+^ cases were also HPV16 E7 mRNA^+^. The proportion of HPV positivity was higher in male patients (n = 47, 69.1%) than in female (n = 21, 30.9%). The HPV^+^ cases in male patients were 27 (39.7%) that tested hrHPV DNA^+^, while 25 (36.8%) were HPV16 DNA^+^. In contrast, only 10 (14.7%) female patients were hrHPV DNA^+^, with 9 (13.2%) being HPV16 DNA^+^. For hrHPV E7 mRNA positivity, 18 (26.5%) males and 9 (11.8%) females were positive, with similar proportion (88.9% each) for HPV16 E7 mRNA positivity (16 males, 8 females). Unsurprisingly, the prevalence of HPV infection varied by anatomical sites. The positivity of hrHPV DNA was notably higher in OPSCC compared to non-OPSCC, with 38.2% (26/65) vs. 16.2% (11/65). Among OPSCC, 96.2% (25/26) of hrHPV DNA^+^ OPSCC cases were also hrHPV E7 mRNA^+^, with 92.3% (24/36) being HPV16 DNA^+^/E7 mRNA^+^. In contrast, non-OPSCC cases had a lower hrHPV DNA positivity (n = 11, 16.2%), with only one case (1.5%) being hrHPV E7mRNA^+^, which was also HPV16 E7 mRNA^+^.

### 2.3. Discrepancies Between HPV DNA and mRNA Testing

**Figure 1:**
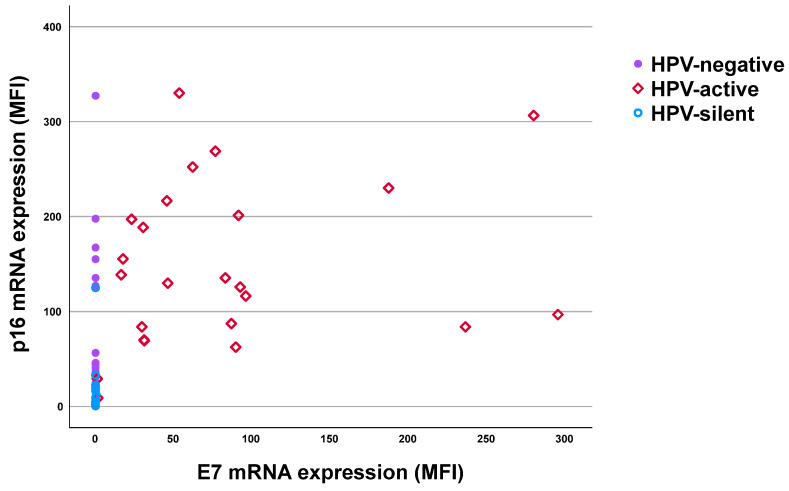
Correlation of p16 mRNA expression with E7 mRNA expression. The normalized MFIs of p16 and E7 mRNA expression using QG-MPH were plotted for each sample. Samples were coded by color for three putative etiologic groups: (a) HPV-active (HPV E7 mRNA^+^ cases, red squares); (b) HPV-silent (HPV DNA^+^ but E7 mRNA^−^ cases, blue circles); (c) HPV-negative (both HPV DNA^−^ and E7 mRNA^−^ cases, purple dots). Abbreviation: MFI = median fluorescence intensity; HPV-active = transcriptionally active HPV infection; HPV-silent = transcriptionally silent HPV infection.

The differences in HPV molecular diagnosis when comparing DNA and mRNA testing are highlighted in Table 4. Consistently, 23 cases were both hrHPV DNA^+^ and mRNA^+^ (hrHPV DNA^+^/mRNA^+^). Interestingly, three cases showed DNA negativity but with E7 mRNA transcription detectable (hrHPV DNA^−^/mRNA^+^). Together, 26 cases were classified as patients with transcriptionally active hrHPV infection based on hrHPV E7 mRNA^+^ (Figure 1, red squares). Additionally, 14 (25.6%) cases were positive for hrHPV DNA but negative for E7 mRNA, indicating the presence of HPV infection but classified as transcriptionally silent (hrHPV DNA^+^/E7 mRNA^−^, Figure 1, blue circles) according to the methodology. At last, 28 (41.1%) cases were negative for both hrHPV DNA and E7 mRNA (Figure 1, purple dots) and classified as HPV-negative tumors.

HPV16 infection was the dominant type among the analyzed HNSCC cases, with 91.9% (34/37) of hrHPV DNA^+^ and 92.3% (24/26) of hrHPV E7 mRNA^+^ being linked to HPV16 in the study. Out of the total, 34 (52.3%) were HPV16 DNA^+^, and HPV16 E7 mRNA^+^ was observed in 24 (36.9%) cases, classified as transcriptionally active HPV16 infections. Among the 34 HPV16 DNA^+^ cases, 13 (38.3%) were E7 mRNA^−^ cases, showing discrepancies in HPV status between Multiplexed Papillomavirus Genotyping (MPG) and QG-MPH measurement and classified as transcriptionally silent HPV16 infections (HPV16 DNA^+^/E7 mRNA^−^). In total, 28 cases (43.1%) were negative for both HPV16 DNA and E7 mRNA and thus classified as HPV16-unrelated etiology (HPV16 DNA^−^/E7 mRNA^−^).

### 2.4. Comparison of p16 mRNA and p16 Protein Expression with HPV Status

To better understand the etiological role of HPV in HNSCC, an in-depth analysis of p16 mRNA and p16 protein expression was conducted in the HPV16-related cases (n = 65). The results were stratified based on HPV DNA and mRNA status.

#### 2.4.1. Correlation of p16 mRNA Expression with HPV16 DNA and E7 mRNA Status

The E7 mRNA^+^ group showed the highest mean MFI of p16 mRNA expression at 132.6 (range 8.9–330.1, Table 5), regardless of HPV DNA status. The distribution is shown by red squares in Figure 1. In the DNA^+^/E7 mRNA^−^ group, the mean MFI of p16 mRNA was lower at 9.0 (range 0.57–125.1), whereas in the DNA^−^/E7 mRNA^−^ group, the mean MFI value of p16 mRNA was 16.8 (range 0–327.2). Notably, the p16 mRNA expression was significantly higher in the E7 mRNA^+^ group compared to both the DNA^+^/E7 mRNA^−^ group (*p* < 0.001) and the DNA^−^/E7 mRNA^−^ group (*p* < 0.001). Another finding was that the p16 mRNA expression was also lower in the DNA^+^/E7 mRNA^−^ group than that of the DNA^−^/E7 mRNA^−^ group (*p* = 0.050), which might be due to HPV-independent pathway affecting the DNA^−^/E7 mRNA^−^ cases.

#### 2.4.2. Correlation Between p16 Protein Expression and HPV16 DNA and E7 mRNA Status

The correlation between p16 protein expression and HPV16 status is summarized in Table 6. As defined, 79.2% cases (19/24 HPV16 E7 mRNA^+^) presented p16 IHC^+^, while 55.9% cases (19/34 HPV16 DNA^+^) presented p16 IHC^+^, indicating better concordance between overexpressed p16 protein and HPV16 E7 mRNA^+^ status compared to that between p16 protein and HPV16 DNA^+^ status.

When relating the p16 IHC negativity to HPV status, the majority of p16 IHC^−^ cases were found in groups without transcriptionally active HPV infection. In summary, 37 out of 41 HPV mRNA^−^ cases (90.2%) were p16 IHC^−^, while 28 out of 31 HPV DNA^−^ cases (90.3%) were p16 IHC^−^. Noteworthy, in the HPV16 DNA^+^/mRNA^−^ group, 12 out of 13 cases (92.2%) were p16 IHC^−^, plus one p16 IHC-invalid case. The results also showed that 28 HPV DNA^−^ cases were correctly interpreted as p16 IHC^−^, while 37 HPV mRNA^−^ cases were p16 IHC^−^, highlighting that p16 protein expression was more closely associated with transcriptionally active HPV (as indicated by E7 mRNA status) than with HPV DNA testing alone. The concordance between HPV mRNA and p16 IHC was higher than that between HPV DNA and p16 IHC.

### 2.5. Impact of HPV Status and p16 IHC Status on Prognosis

**Figure 2:**
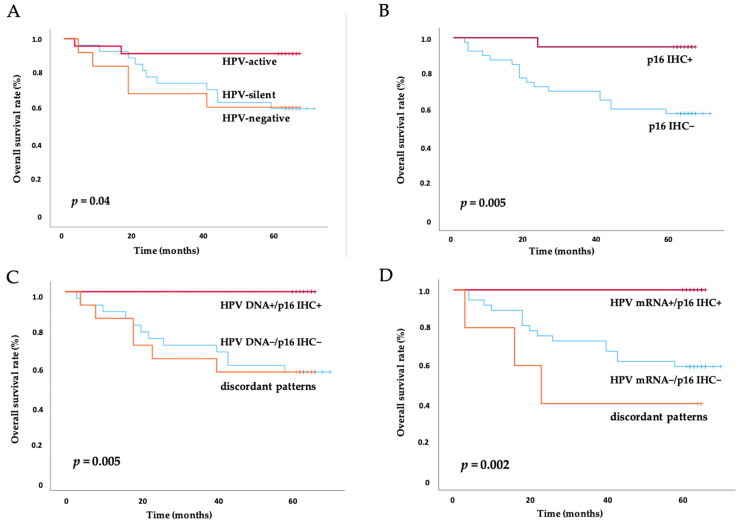
HNSCC patients’ survival disparities related to HPV status or p16 IHC status. (**A**) Impact of HPV transcriptional status on OS rates (*p* = 0.04); (**B**) impact of p16 IHC status on OS (*p* = 0.005); (**C**) impact of HPV DNA status in combination with p16 IHC status on OS rates (*p* = 0.005). Discordant patterns were identified as mismatches between either HPV DNA testing or p16 IHC results; (**D**) impact of HPV mRNA status in combination with p16 IHC on OS rates (*p* = 0.002). Discordant patterns were identified as mismatches between either HPV mRNA testing or p16 IHC results. The *p*-values displayed in this Figure are the overall differences in survival across groups using Kaplan–Meier analysis and evaluated with a log-rank test. Abbreviation: OS = overall survival. HPV-active = transcriptionally active HPV infection; HPV-silent = transcriptionally silent HPV infection. The p16 protein expression by immunohistochemistry staining was interpreted as positive (p16 IHC+) and negative (p16 IHC^−^).

The prognostic impact of HPV DNA and E7 mRNA status and p16 IHC were investigated, particularly focusing on the transcriptional activity of the HPV, as indicated by E7 mRNA expression, and the corresponding survival disparities (Figure 2).

A statistically significantly better overall survival (OS) rate was demonstrated in the transcriptionally active HPV-infected (E7 mRNA^+^) group compared to those with transcriptionally silent (DNA^+^/E7 mRNA^−^) or HPV-negative (DNA^−^/E7 mRNA^−^) cancers (overall *p* = 0.04 at log-rank, Figure 2A). Specifically, 91.7% (22/24) of the patients in the E7 mRNA^+^ group were still alive at the last 5-year follow-up, compared to a lower OS rate in the transcriptionally silent group (61.5%, 8/13, *p* = 0.032 at log-rank) and the HPV-negative group (60.7%, 17/28, *p* = 0.016 at log-rank) and similarity in poorer outcome between the latter two groups (*p* = 0.873 at Log-rank).

As expected, p16 IHC^+^ was closely associated with a significantly better prognosis compared to p16 IHC^−^ (*p* = 0.005 at log-rank, Figure 2B). At the 5-year follow-up, 95.0% (19/20) of patients with p16 IHC^+^ were alive, compared to only 58.5% (24/41) of those with p16 IHC^−^.

The prognostic implications of combined HPV DNA or HPV mRNA status with p16 IHC was analyzed, refining the ability to predict patient outcomes. Patients who tested both HPV DNA^+^ and p16 IHC^+^ had a significantly better OS rate compared to those who were HPV DNA^−^ and p16 IHC^−^ or had discordant patterns (overall *p* = 0.005 at log-rank, Figure 2C). Remarkably, 100% (19/19) of patients in the HPV DNA^+^/p16 IHC^+^ group were alive at the 5-year follow-up, whereas only 57.1% (16/28) of patients in the HPV DNA^−^/p16 IHC^−^ group survived (*p* < 0.001 at log-rank), and 57.1% (8/14) of those with discordant patterns survived (*p* < 0.001 at log-rank).

In addition, 100% (19/19) of the patients in the HPV mRNA^+^/p16 IHC^+^ group exhibited significantly better survival during the 5-year follow-up (overall *p* value =0.002 at log-rank, Figure 2D), compared to 59.5% (22/37) in the HPV mRNA^−^/p16 IHC^−^ group (*p* = 0.001 at log-rank) and 40.0% (2/5) in the discordant group (*p* < 0.001 at log-rank), each group separately. Further, the results also identified that patients with discordant expression patterns, either between HPV DNA status and p16 IHC status, or between HPV mRNA and p16 IHC status, had worse outcomes. Specifically, the OS rate was 57.1% (8/14) in the group with discordant HPV DNA and p16 IHC results, compared to 40% (3/5) in the group with discordant HPV E7 mRNA and p16 IHC results, indicating the prognostic disadvantage for patients with discordant categories and the advantages for incorporating the molecular mRNA profiling by QG-MPH to p16 IHC in making therapeutic decision and predicting patient outcomes in HNSCC.

Additionally, the correlation between p16 mRNA expression and p16 IHC status was analyzed (Supplementary Figure S1). The p16 mRNA expression was significantly higher in the p16 IHC^+^ group (mean MFI 127.8, range 31.0–330.1), as compared to the p16 IHC^−^ group (mean MFI 12.2, range 0.6–327.2). Interestingly, 19.5% (8/41) of the p16 IHC^−^ cases showed notably elevated p16 mRNA expression, ranging from 125.1 to 327.2. These outliers with increased p16 mRNA expression survived for more than 5 years, suggesting that elevated p16 mRNA expression might be indicative for a better prognosis, even in the absence of detectable p16 protein by IHC.

**Figure 3:**
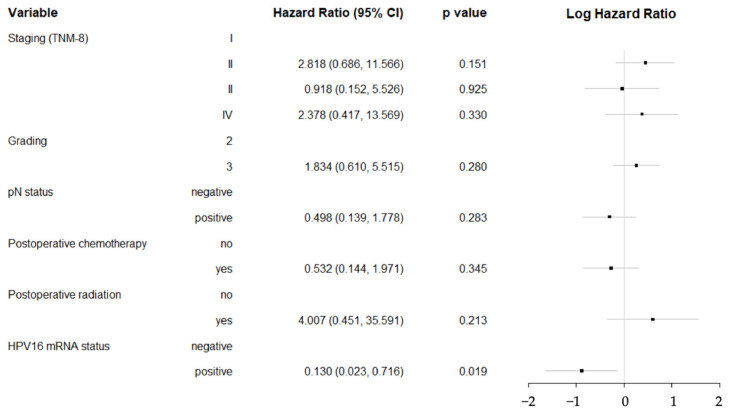
Forest plot showing hazard ratios for clinicopathologic characteristics. 5-year survival was analyzed using multivariate Cox regression in patients with HPV16-associated HNSCC. Only HPV16 mRNA positivity emerged as an independent factor associated with a significantly lower risk (*p* = 0.019). The black dots represent the log-transformed hazard ratios, and the horizontal lines indicate the log-transformed 95% confidence intervals (95% CI).

A multivariate Cox regression model further identified that staging, tumor grading, pN status, and receiving postoperative chemo-/radiotherapy were not significant risk factors (Figure 3). Only HPV16 mRNA positivity emerged as an independent factor associated with a significantly lower risk (*p* = 0.019).

## 3. Discussion

The prognosis of HNSCC varies widely depending on the etiology, with HPV-driven cases generally having a better outcome than HPV-unrelated ones [5]. Traditionally, HPV status is tested through HPV DNA, and p16 overexpression has been suggested as a surrogate marker for HPV status in the TNM-8 system [13,20]. However, exceptions exist. Cases with discordant patterns, such as HPV DNA^−^/p16 IHC^+^ or HPV DNA^+^/p16 IHC^−^, are associated with worse outcomes [5,16]. This highlights the limitations of relying solely on HPV DNA testing alone or in combination with p16 IHC. Our study underlines the limitations of DNA-based HPV testing plus p16 IHC. The findings pointed out that within the HPV DNA^+^ HNSCC cases, not only the molecular profiles but also patients’ prognosis differ significantly based on HPV mRNA expression status. Transcriptionally active HPV oncogene expression is a necessary activity of truly HPV-driven HNSCCs [5]. Therefore, precise and accurate etiological identification of transcriptionally active HPV infection is essential, as only these represent true HPV-driven cancers, facilitating the optimization of individual personalized therapeutic strategies.

We describe here the use of the QG-MPH assay to profile HPV oncogene E7 and p16 ^INK4a^ mRNA expression, enhancing the accuracy of HPV etiological diagnosis. One of the most relevant results of our research was that 54.4% of cases tested hrHPV DNA^+^, while only 38.2% expressed detectable hrHPV E7 mRNA levels, indicating active infections. HPV E7 mRNA profiling enables more assured molecular diagnosis by distinguishing transcriptionally active from silent HPV infections. Notably, we found that HPV16 was most commonly transcriptionally active in OPSCC, while non-OPSCC cases were found with transcriptionally silent HPV infections, explaining their worse prognosis [5,21]. These findings align with previous reports [6,16,22,23,24].

The truly HPV-driven mechanism in HNSCC is primarily mediated by the expression of viral oncogene E6 and E7 (see Supplementary Figure S2). E7 drives cancer cell proliferation by binding to the retinoblastoma protein (pRB), a key cell-cycle regulator, leading to the release of the transcription factor E2F, resulting in unscheduled cell-cycle progression and enhanced cancer cell proliferation. E7 also causes aberrant compensatory upregulation of p16 ^INK4a^, followed by overexpression of the p16 protein [25,26]. Consistent with a previous report [10], our results show that transcriptionally active HPV infections (E7 mRNA^+^) induce significant p16 mRNA upregulation, which, in turn, causes higher expression of p16 protein (p16 IHC^+^), when compared to HPV DNA^+^/E7 mRNA¯ and HPV-negative cases. Additionally, E6 induces the degradation of functional wild-type p53, thereby impairing its tumor suppressing functions, such as growth-arrest and inducing apoptosis [27]. While E6-mediated degradation of wild-type p53 may affect p53 functions less than p53 mutations itself [10], apoptotic pathways can still be activated upon exposure to radiation with functional p53 [9,27]. This explains why patients with truly HPV-driven malignancies have better outcomes, as substantiated by survival analysis in the current study. In contrast, we observed that most HPV DNA^+^ but E7 mRNA¯ cases did not show any upregulation of p16 mRNA or overexpression of p16 protein, further supporting that the transcriptionally silent HPV-infected cases should be classified as pseudo-HPV-driven cancers. Along with HPV-negative HNSCC, these cancers should be considered HPV-unrelated. Next-generation sequencing studies have shown that p53 mutations are present in 75–85% HPV-unrelated HNSCC, initiated by other risk factors [9,28]. Dysfunctional p53-mutations disable cell-cycle arrest and apoptosis, resulting in cell survival and resistance to radiation [9], which aligns with our findings of the clinical data that HPV-unrelated patients have a poorer outcome.

We next evaluated the prognostic value of combining HPV mRNA status and p16 IHC on prognosis in terms of 5-year OS. Patients with transcriptionally active HPV infections (HPV DNA^+^/E7 mRNA^+^) had significantly higher OS rate compared to those with transcriptionally silent (HPV DNA^+^/E7 mRNA¯) or HPV-negative tumors. Furthermore, we found that patients in the HPV E7 mRNA^+^/p16 IHC^+^ group had significantly better outcomes than those in the HPV E7 mRNA^−^/p16 IHC^−^ group. Specifically, the poorest outcomes were observed in patients with discordant HPV DNA and p16 IHC results in the study. It is attractive to argue that these patients may have been misclassified and that future patients with discordant status should be treated more appropriately, avoiding unfavorable outcomes and shorter survival. In a previous report, patients with discordant testing patterns (HPV DNA^+^/p16 IHC^−^ or HPV DNA^−^/p16 IHC^+^) had a significantly worse prognosis than patients testing HPV DNA^+^/p16 IHC^+^, which is consistent with our findings [29], and highlights the need for comprehensive molecular profiling [29].

Misclassification of discordant cases can lead to suboptimal treatment strategies and significantly impact prognosis. For example, HPV DNA^+^/E7 mRNA^−^/p16 IHC^−^ cases suggest transcriptionally silent HPV infection, indicating the tumor was likely induced by non-HPV etiological factors. Such cases resemble HPV-unrelated HNSCC in behavior, and treatment as HPV-driven HNSCC can lead to inappropriate de-escalation of therapy, resulting in undertreatments and increase the risk of recurrence and poorer outcomes. Moreover, HPV DNA^−^/E7 mRNA^−^/p16 IHC^+^ cases may represent an unknown, non-HPV-related etiological pathway, leading to a pseudo-HPV-driven HNSCC that mimics the p16 upregulation seen in HPV-driven cancers. Treating these cases as HPV-driven could lead to underestimation of aggressiveness, resulting in insufficiently intense treatment and adverse outcome. Our findings confirm that integrating HPV E7 mRNA testing using QG-MPH, rather than the HPV DNA testing, identifies HPV as a causal factor in carcinogenesis. This approach significantly improves the detection of transcriptionally active HPV-driven tumors, leading to more accurate diagnoses and prognosis prediction for HNSCC patients. Consequently, it has the potential to refine personalized therapeutic strategies and improve patient outcomes.

A limitation of our study is the use of a formalin-fixed-paraffin-embedded (FFPE) material stored for more than 15 years. The possibility of potential degradation of DNA and mRNA in these FFPE blocks should be considered. Another limitation is sample size by different anatomic sites. Our observations need therefore to be confirmed on a larger cohort of patients. However, the corresponding results of etiological classification and the Kaplan–Meier survival analysis provide solid support to the meaningfulness of the data. Fresh tumor tissue samples should be used in future prospective studies and when actual clinical diagnoses are to be conducted.

In the present work, a major practical advantage is that the novel QG-MPH assay enables an easy and comprehensive measurement of a broad panel of biomarkers, for example, HPV E7 mRNA of 18 individual genotypes plus p16 mRNA, and additional biomarkers, when necessary, within a single analysis, indicating whether the HPV infection is transcriptionally active. Importantly, the QG-MPH assay enhances HPV etiological diagnosis to molecularly distinguish truly HPV-driven HNSCC from truly non-HPV induced malignancies. The potential benefit of the QG-MPH assay is a reduction in misclassification and providing valuable implications for therapeutic decision-making and prognosis.

## 4. Materials and Methods

### 4.1. Clinical Specimens

**Figure 4:**
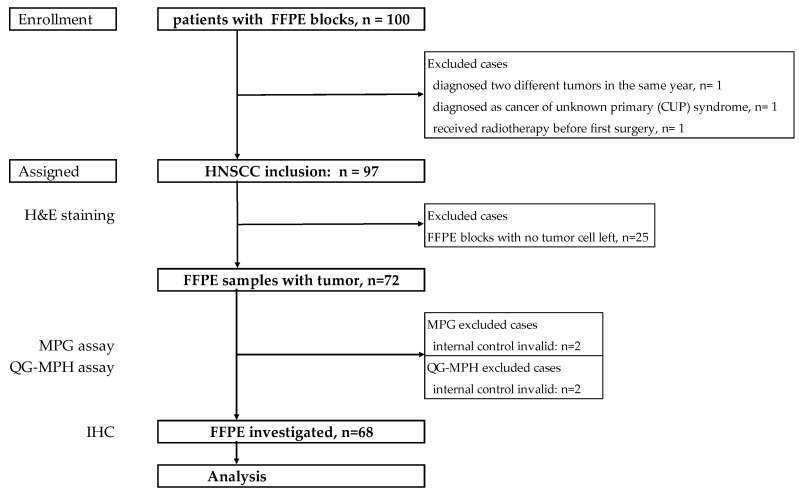
Selection of FFPE specimens from HNSCC patients for the biomarker expression study using QG-MPH. Out of 100 HNSCC cases, 97 were assigned. The specimens were tested by H&E staining for tumor tissue residues. A total of 25 cases were excluded due to non-sufficient tumor tissue. MPG and QG-MPH assays were performed with FFPE sections of 72 specimens, and 68 were valid for further analysis. Abbreviation: FFPE = formalin-fixed-paraffin-embedded; HNSCC = head and neck squamous cell carcinoma; H&E staining = hematoxylin and eosin staining; MPG = Multiplex Papillomavirus Genotyping assay; QG-MPH = QuantiGene-Molecular-Profiling-Histology assay; IHC = immunohistochemistry.

The Institutional Review Board of the Charité-Universitätsmedizin Berlin, Germany (EA4/035/08) approved this study. Patient’s clinical characteristics, including age, gender, and clinical and pathological diagnosis, were retrieved from medical records. The key eligibility criterion was pathologically confirmed HNSCC at initial diagnosis. FFPE blocks were provided by the Pathology Department of Ernst von Bergmann Clinic, a Charité-Universitätsmedizin Berlin-affiliated hospital in Potsdam, Germany. In total, 227 patients diagnosed as HNSCC with clinical data were obtained and analyzed, as shown in a previous publication [30]. For this study, a total of 100 FFPE blocks were available from those patients whose specimens were collected at the surgery after first diagnosis and archived between 2001 and 2013 (Figure 4). Three cases were excluded when censoring the medical history, including one patient who was diagnosed with two different primary tumors, one cancer of unknown primary, and one treated with radiation before salvage surgery.

### 4.2. Molecular Slicing and Histopathological Evaluation of FFPE Blocks

To exclude carry-over between patients’ materials for molecular analyses, the microtome was thoroughly cleaned with DNA/RNA-ExitusPlus ™ (PanReac AppliChem, Cat. # A7089, Darmstadt, Germany) between different patient’s FFPE blocks and before the start of molecular sectioning. For each sample, a new disposable blade was used. FFPE blocks of mouse liver were sectioned after cleaning and prior to each patient’s specimen and analyzed for HPV genotypes and human actin-β negativity. Each FFPE block was serially sectioned using a sandwich method. The first and last slides (2–5 μm thickness) were used for histopathological verification after hematoxylin and eosin (H&E) staining. Two pathologists confirmed the presence of tumor cell residues and estimated the percentage of tumor cell area. The blocks with less than 5% area of tumor cells were excluded from the analysis, determined as nonsufficient tumor tissue. The average tumor cell percentage (TCP) area was calculated by the formula:

$$Average\;TCP=\left(TCP1\;\mathrm{in}\;\mathrm{the}\;\mathrm{first}\;\mathrm{slide}+TCP2\;\mathrm{in}\;\mathrm{the}\;\mathrm{last}\;\mathrm{slide}\right)÷2$$

In-between, two sets of sections (5–10 μm thickness, 5–10 slices) were collected in DNA/RNAase-free microcentrifuge tubes and tested for HPV DNA and mRNA using MPG and QG-MPH assays, respectively.

### 4.3. DNA Extraction and Multiplexed Papillomavirus Genotyping (MPG) Assay

DNA extraction and purification from one set of FFPE sections was conducted by Maxwell^®^ 16 FFPE Plus LEV DNA purification kit (Promega, Cat. #AS1135, Madison, WI, USA) according to the standard protocol. HPV genotyping was performed and analyzed using an MPG assay, as described in prior studies 18, 19. The MPG assay has analytical sensitivity to detect eighteen hrHPVs (HPV16, 18, 26, 31, 33, 35, 39, 45, 51, 52, 53, 56, 58, 59, 66, 68, 73, 82) and nine low-risk HPVs (HPV6, 11, 42, 43, 54, 57, 70, 72, 90) by amplification of a generic-L1 gene sequence and subsequent genotyping enabled by genotype-specific probes conjugated to Luminex-suspension beads. Negative and positive controls and a human β-globin internal control monitored the sample cellularity, the efficiency of DNA isolation, PCR amplification, hybridization, and genotyping. The median fluorescence intensity (MFI) of probe signal had to be three-fold higher than the MFI of the background to count as positive. None of the mouse liver control samples tested positive for HPV or human β-globin DNA, confirming the absence of cross-contamination between patients FFPE sections.

### 4.4. RNA Extraction and QuantiGene-Molecular-Profiling-Histology (QG-MPH) Assay

RNA was released from FFPE sections (5 μm thickness, 5–10 pieces) using the QuantiGene FFPE Sample Processing Kit (ThermoFisher, Cat. #QS0109, Waltham, MA, USA) according to the manufacturer’s protocol. Briefly, the sections were incubated with 200 µL of a homogenizing buffer supplemented with 3.3 µL of proteinase K solution (50 µg/µL) in the microfuge tubes for 12–18 h at 65 °C at 650 rpm in a Thermoshaker. The samples were centrifuged for 5 min at 10,000× *g* (Microfuge, Heraeus, Hanau, Germany) at room temperature. The aqueous lysate containing the desired RNA was collected and transferred to a MultiScreen Filter plate (Merck Millipore, Dublin, Ireland). The filter plate was then placed on top of another clean 96-well plate and centrifuged (Multifuge 1S-R, Heraeus, Hanau, Germany) for 5 min at 2500 rpm to remove any paraffin residuals. The crude lysate was then ready for downstream QG-MPH assay or stored at −80 °C for later use.

The QG-MPH Kit (ThermoFisher, Waltham, MA, USA) contains a panel of custom oligonucleotides for mRNA capture hybridization and quantification assay based on Luminex suspension bead array multiplexing and branched DNA signal amplification. The QG-MPH diagnostic assay comprises the multiplexed detection of eighteen hrHPV (HPV16, 18, 26, 31, 33, 35, 39, 45, 51, 52, 53, 56, 58, 59, 66, 68, 73, 82) E7 oncogene mRNA, three spliced HPV16 mRNAs (16E1_4, 16E6*I and 16E1C), and cellular biomarker mRNAs (like p16 ^INK4a^, Ki67, p53, and others) in a custom plex set [31]. These are informative about the exact HPV genotype-specific E7 mRNA status and the severity of cellular transformation. On each plate, three negative control (NC) wells containing a plex set and capture-bead mixture plus buffer only and one lysate of the HPV16-positive cervical cancer cell line CaSki as a positive control (PC) were included. The ACTB mRNA served as the internal control and for normalization to cellularity.

As described previously with some modification [31], 20 µL of the FFPE extraction lysate was applied to a well of a 96-well hybridization plate and mixed with 15 µL of the premixture containing the probe set, capture beads, blocking reagent, and proteinase K, according to the manufacturer’s protocol. For capture hybridization of specific target mRNAs, the plate was sealed and incubated for 18–22 h at 54 °C, 600 rpm in a microplate thermostatic shaker (neoLab Migge, Heidelberg, Germany). For signal amplification by branched DNA technology, pre-amplifier, amplifier, biotinylated label probe, and streptavidin–phycoerythrin (SAPE) were sequentially incubated according to the protocol. MFI was detected by a Bioplex 200 instrument (BioRad, Hercules, CA, USA) and recorded per bead class, i.e., mRNA target.

For HPV genotyping by genotype-specific E7 mRNA, the cutoff was set at the mean of the specific bead class background plus 3 × standard deviation (SD) of the NK:

cutoff=mean+3×SD

The sample was considered positive when the detected MFI was higher than the cutoff value, accordingly.

For the quantification of relative p16 mRNA expression (referred to herein as p16 mRNA), the MFI value in reactions without the sample lysate was determined as p16 NC. The net MFI values of p16 and ACTB were calculated by subtracting the average NC from the raw value; e.g., the net MFI value of p16 was equal to the raw p16 MFI minus the mean p16 MFI of the NC. Furthermore, the MFI of the relative p16 mRNA expression that was normalized by the ACTB of the individual sample was calculated by the following formula:

$$\mathrm{relative}\;p16\;\mathrm{mRNA}\;\mathrm{expression}=\frac{\left(\mathrm{raw}\;p16-\mathrm{mean}\;p16\;\mathrm{of}\;\mathrm{NC}\right)}{\left(\mathrm{raw}\;\mathrm{ACTB}-\mathrm{mean}\;\mathrm{ACTB}\;\mathrm{of}\;\mathrm{NC}\right)\times \mathrm{average}\;\mathrm{TCP}}\times 100$$

### 4.5. Immunohistochemistry

FFPE sections of 2–5 μm thickness were stained on a Ventana BenchMark ULTRA (Roche Tissue Diagnostics, Basel, Switzerland) using antibodies against p16 (clone E6H4, solution 1:2, Ventana) following the manufacturer’s protocol. Two well-trained pathologists, Eliane Taube and Jonathan Pohl, assessed the p16 IHC. The p16 staining was interpreted as block positivity/diffuse when strong nuclear or strong nuclear and cytoplasmic staining of a field of tumor cells was identified. The result was marked as failed when there were insufficient tumor cells in the slide and negative when only individual interspersed cells were stained.

### 4.6. Statistical Analysis

Statistical analyses were carried out using SPSS 29.0 (SPSS Inc., Chicago, IL, USA). Categorical variables were presented as absolute numbers and percentages. The MFI was described as median value and range (from minimum to maximum value). The statistical analyses compared categorical parameters using a Chi-square or Fisher’s exact test. The Pearson’s Chi-square test or Fisher’s exact test was used to compare quantitative independent variables, while for nonparametric variables, a Mann–Whitney U test was used. The length of survival (months) was calculated from the date of first diagnosis to the date of death or last survival follow-up 5 years later; the overall survival was calculated via a Kaplan–Meier analysis, and significance was evaluated with the log-rank test. *p*-values were calculated excluding missing values and were considered statistically significant when *p* < 0.05.

## 5. Conclusions

The innovative QG-MPH assay offers a multiplexed and accurate measurement of the HPV E7 and p16 mRNA in HNSCC specimen and enables more precise differentiation between truly HPV-driven HNSCC and transcriptionally silent, non-etiological HPV infections. Thereby, the assay has the potential to strengthen the accuracy of HPV etiological diagnosis, ultimately leading to better clinical outcomes.

## Figures and Tables

**Table 1:** Clinical characteristics.

Characteristics	N = 68 (100%)
Age (years) at Diagnosis	
Mean ± standard deviation	60.8 ± 10.1
Gender	
Male	47 (69.1%)
Female	21 (30.9%)
Body Mass Index (BMI)	
<18.5	2 (2.9%)
18.5–24.9	28 (41.2%)
25.0–29.9	25 (36.8%)
>30.0	8 (11.8%)
missing	5 (7.4%)
Smoking history	
No	12 (17.6%)
Yes	48 (70.6%)
missing	8 (11.8%)
Alcohol consumption history	
No	28 (41.2%)
Yes	16 (23.5%)
missing	26 (38.2%)
Anatomic site	
Anterior tongue	8 (11.8%)
Base of tongue	11 (16.2%)
Tongue (undefined)	4 (5.9%)
Floor of the mouth	14 (20.6%)
Oropharynx (undefined)	2 (2.9%)
Retromolar trigone	2 (2.9%)
Soft palate	2 (2.9%)
Tonsil	25 (36.8%)
Anatomic subgroup	
OPSCC	40 (58.8%)
non-OPSCC	28 (41.2%)
Staging (AJCC, 8th)	
I	16 (23.5%)
II	26 (38.2%)
III	12 (17.6%)
IV	14 (20.6%)
Grading	
I	2 (2.9%)
II	33 (48.5%)
III	33 (48.5%)
IV	0

Abbreviation: OPSCC indicates oropharyngeal squamous cell carcinoma, including tumors arising from the base of tongue, tonsils, oropharynx (undefined), and soft palate, while the remainders are grouped as non-oropharyngeal squamous cell carcinoma (non-OPSCC). AJCC, 8th = American joint committee on cancer, 8th Edition.

**Table 2:** Clinicopathological features stratified by HPV16 mRNA status.

Characteristics		HPV16 mRNA Status		*p*-Value
		Positive, n = 24	Negative, n = 41	
Age, mean ± standard deviation		63.54 ± 9.77	59.49 ± 10.45	0.128
Staging (TNM-8)				
	1	5 (20.8)	11 (26.8)	0.010
	2	15 (62.5)	9 (22.0)	
	3	2 (8.3)	9 (22.0)	
	4	2 (8.3)	12 (29.3)	
Tumor Grade				
	2	9 (37.5)	22 (56.4)	0.195
	3	15 (62.5)	17 (43.6)	
			missing, n = 2	
Pathological nodal (pN) status				
	negative	9 (37.5)	17 (41.5)	0.798
	positive	15 (62.5)	24 (58.5)	
			missing, n = 2	
Postoperative radiation, n (%)				
	yes	16 (66.7)	36 (87.8)	0.056
	no	8 (80.8)	5 (12.2)	
Postoperative chemotherapy, n (%)				
	yes	7 (29.2)	28 (68.3)	0.004
	no	17 (70.8)	13 (31.7)	

**Table 3:** HPV prevalence of the primary tumor lesion by sex and anatomic sites.

HPV Prevalence		n (%)	HPV DNA Status *			HPV mRNA Status *		
			HPV^−^	hrHPV^+^	HPV16^+^	HPV^−^	hrHPV^+^	HPV16^+^
**in total**		68 (100)	31 (45.6)	37 (54.4)	34 (50.0)	42 (61.8)	26 (38.2)	24 (35.3)
by sex	Male	47 (69.1)	20 (29.4)	27 (39.7)	25 (36.8)	29 (42.7)	18 (26.5)	16 (23.5)
	Female	21 (30.9)	11 (16.2)	10 (14.7)	9 (13.2)	13 (19.1)	8 (11.8)	8 (11.8)
by sites	OPSCC	40 (58.8)	14 (20.6)	26 (38.2)	24 (35.3)	15 (22.1)	25 (36.8)	23 (33.8)
	Non-OPSCC	28 (41.2)	17 (25.0)	11 (16.2)	10 (14.7)	27 (39.7)	1 (1.5)	1 (1.5)

* When the internal control (beta-globin for L1 DNA and actin-beta for E7 mRNA) is valid and the detection of L1 DNA or E7 mRNA is greater than the cutoff, the HPV status is determined as positive (+); otherwise, it is negative (−). Abbreviation: hrHPV = high-risk Human Papillomavirus; OPSCC = oropharyngeal squamous cell carcinoma.

**Table 4:** HrHPV and HPV16 prevalence by HPV DNA and E7 mRNA status.

hrHPV mRNA	hrHPV DNA *			HPV16 mRNA	HPV16 DNA **		
	Positive	Negative	in Total		Positive	Negative	in Total
**positive**	23	3	26	**positive**	21	3	24
**negative**	14	28	42	**negative**	13	28	41
**in total**	37	31	68	**in total**	34	31	65

* The HPV status is presented in relation to HPV DNA and mRNA detection. Discrepancies between HPV DNA and mRNA positivity are listed in this Table. ** In addition to HPV16, hrHPV18, 33, and 82 were detected in three individual specimens (data not included in this Table). Abbreviation: hrHPV = high-risk Human Papillomavirus.

**Table 5:** Comparison of p16 mRNA expression by different HPV etiological roles.

p16 mRNA Expression *	DNA^+/−^	DNA^+^	DNA^−^	*p*-Value ^(1)^	*p*-Value ^(2)^	*p*-Value ^(3)^
	**E7 mRNA^+^**	**E7 mRNA^−^**	**E7 mRNA^−^**			
**Samples (n)**	24	14	28			
**Mean MFI**	132.6	9.0	16.8	<0.001	<0.001	0.050
**Range**	8.9–330.1	0.6–125.1	0.0–327.2			

* The p16 mRNA expression, recorded in median fluorescent intensity (MFI), was normalized per specimen and calculated mean value for each group. ^(1)^ Comparison of relative p16 mRNA expression between truly HPV-driven (E7 mRNA^+^, irrespective of DNA detection) cases and transcriptionally silent HPV-infected (DNA^+^/E7 mRNA^−^) cases. ^(2)^ Comparison of relative p16 mRNA expression between HPV-driven cases and HPV-negative (DNA^−^/E7 mRNA^−^) cases. ^(3)^ Comparison of relative p16 mRNA expression between transcriptionally silent HPV-infected cases and HPV-negative cases.

**Table 6:** Correlation between p16 protein expression and HPV16 status.

HPV16 Status *	n	p16 IHC (n)		
		Positive	Negative	Invalid **
**DNA^+^/mRNA^+^**	21	19	1	1
**DNA^−^/mRNA^+^**	3	0	3	0
**DNA^+^/mRNA^−^**	13	0	12	1
**DNA^−^/mRNA^−^**	28	1	25	2

* The HPV status is presented as positive (+) and negative (−) by combining the results of both HPV16 DNA and mRNA testing. The p16 protein expression by IHC was interpreted as positive and negative. ** Due to insufficient tumor tissue for IHC interpretation, the four cases were marked as invalid. Abbreviation: IHC = immunohistochemistry staining.

## Data Availability

The original contributions presented in this study are included in the article/Supplementary Materials. Further inquiries can be directed to the corresponding author.

## Supplementary Materials

The following supporting information can be downloaded at https://www.mdpi.com/article/10.3390/ijms252413643/s1.
