# Supplementary material for: Identifying Truly HPV-Driven Head and Neck Squamous Cell Carcinoma by QuantiGene-Molecular-Profiling-Histology Assay Allows for More Precise Prognosis Prediction
Source: Int J Mol Sci. 2024 Dec 20;25(24):13643. doi: 10.3390/ijms252413643 (PMC11728353; doi:10.3390/ijms252413643)
Supplement: Supplementary file 1 [file ijms-25-13643-s001.zip › Figure S1.pdf]

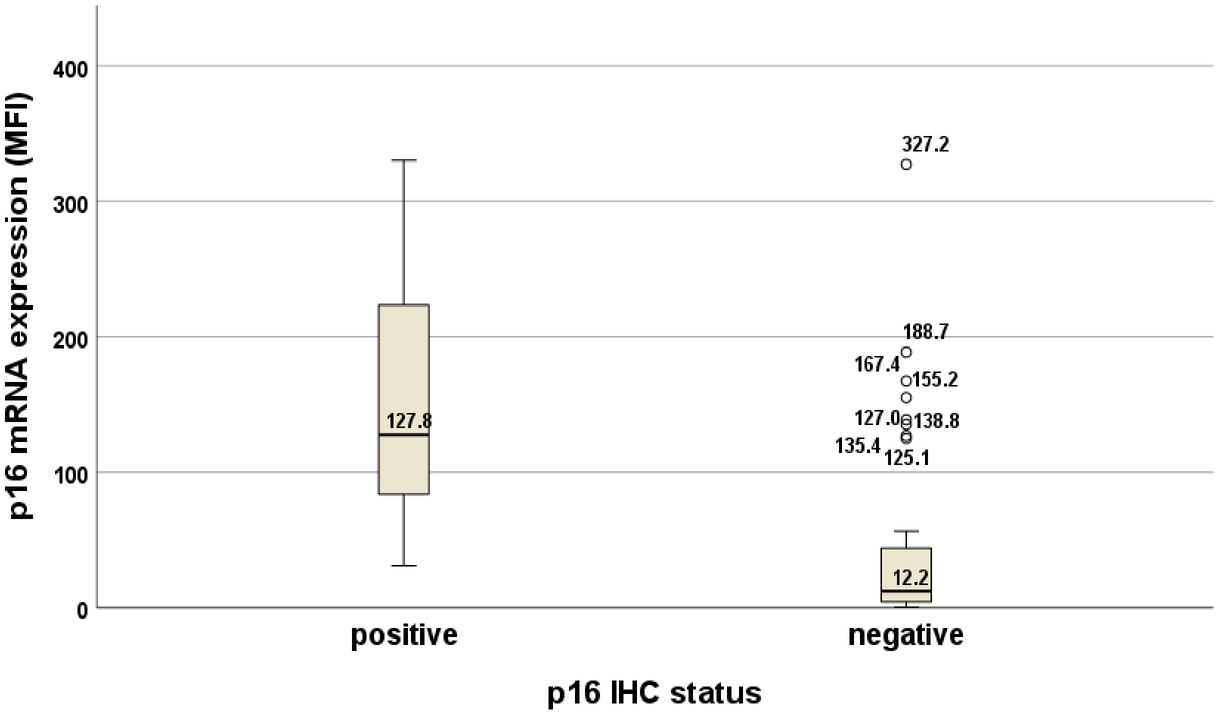

Supplementary Figure S1. Correlation of p16 mRNA expression and p16 IHC status in HNSCC. The mean MFI of p16 mRNA was 127.8 (range 31.0 - 330.1) in the p16 IHC-positive group and 12.2 (range 0.6 - 327.2) in the p16 IHC-negative group. The outliers (19.5%, 8/41) in the p16-IHC negative group was indicated by black circles and their MFI, respectively. These outlier patients were found with increased p16 mRNA expression and survived for more than 5 years.
