# Supplementary material for: Identifying Truly HPV-Driven Head and Neck Squamous Cell Carcinoma by QuantiGene-Molecular-Profiling-Histology Assay Allows for More Precise Prognosis Prediction
Source: Int J Mol Sci. 2024 Dec 20;25(24):13643. doi: 10.3390/ijms252413643 (PMC11728353; doi:10.3390/ijms252413643)
Supplement: Supplementary file 1 [file ijms-25-13643-s001.zip › Figure S2. putative mechanism.pdf]

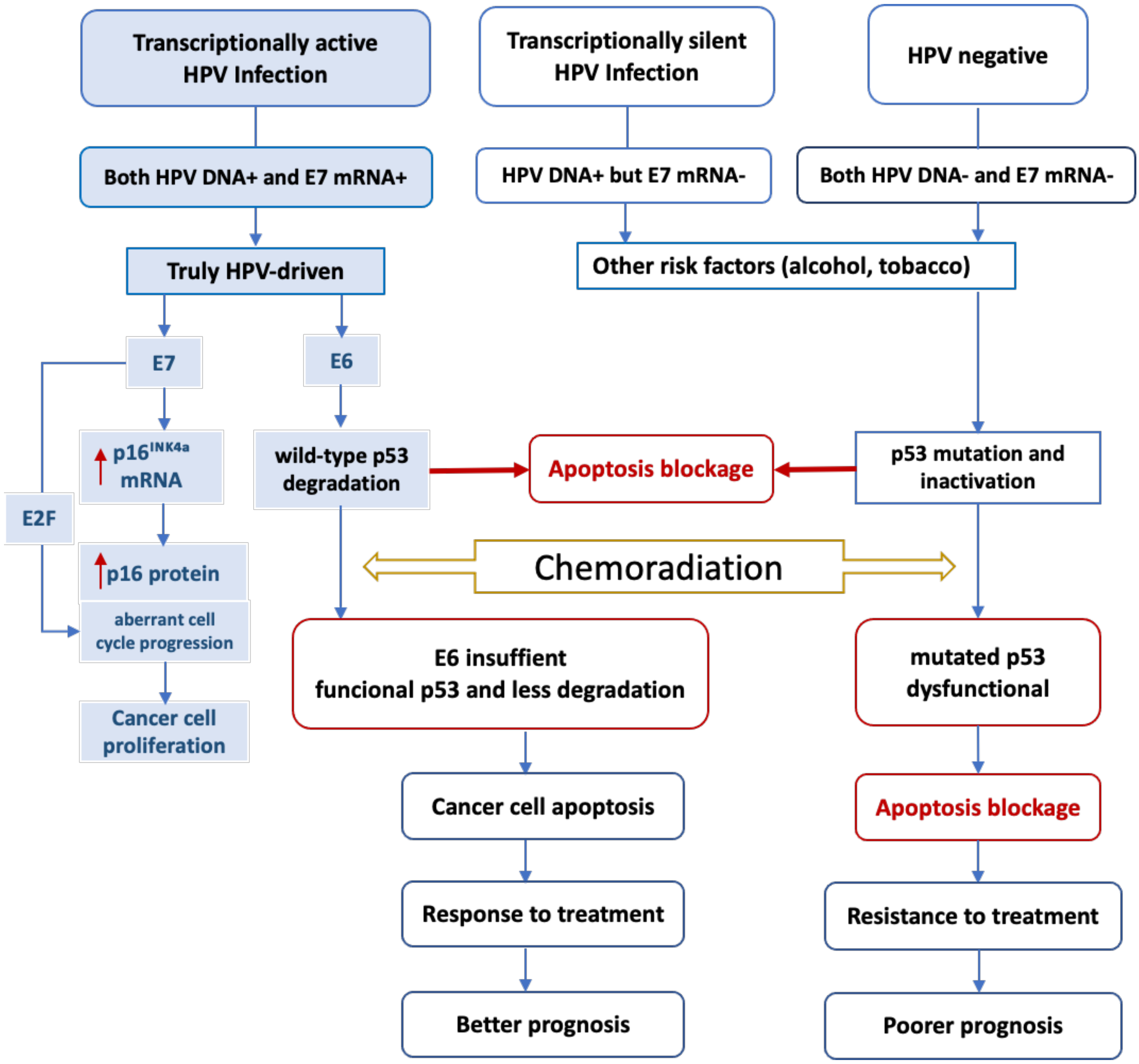

Supplementary Figure S2. Putative mechanism of differential therapy response and outcomes in patients with HPV-driven versus non-HPV-driven HNSCC. The primary difference between these groups lies in the mutational inactivation of p53 in HPV-independent malignancies, which prevents apoptosis induction following chemoradiation therapy. In contrast, in HPV-driven tumors, the E6 viral protein degrades p53 without causing mutational inactivation, leaving the p53 gene functional. This functional p53 enables apoptosis induction in response to chemoradiation, resulting in improved patient outcomes.
